# Discrete typing units of *Trypanosoma cruzi*: Geographical and biological distribution in the Americas

**DOI:** 10.1038/s41597-022-01452-w

**Published:** 2022-06-24

**Authors:** Natalia Velásquez-Ortiz, Giovanny Herrera, Carolina Hernández, Marina Muñoz, Juan David Ramírez

**Affiliations:** 1 Centro de Investigaciones en Microbiología y Biotecnología-UR (CIMBIUR), Facultad de Ciencias Naturales, Universidad del Rosario, Bogotá, Colombia; 2 Centro de Tecnología en Salud (CETESA), Innovaseq SAS, Bogotá, Colombia; 3 Molecular Microbiology Laboratory, Department of Pathology, Molecular and Cell-based Medicine, Icahn School of Medicine at Mount Sinai, New York, NY USA

**Keywords:** Parasite genetics, Parasitic infection

## Abstract

Chagas disease caused by *Trypanosoma cruzi* is a public health issue in Latin America. This highly diverse parasite is divided into at least seven discrete typing units (DTUs) TcI-TcVI and Tcbat. Some DTUs have been associated with geographical distribution in epidemiological scenarios and clinical manifestations, but these aspects remain poorly understood. Many studies have focused on studying the parasite and its vectors/hosts, using a wide variety of genetic markers and methods. Here, we performed a systematic review of the literature for the last 20 years to present an update of DTUs distribution in the Americas, collecting ecoepidemiological information. We found that the DTUs are widespread across the continent and that there is a whole gamma of genetic markers used for the identification and genotyping of the parasite. The data obtained in this descriptor could improve the molecular epidemiology studies of Chagas disease in endemic regions.

## Abstract (summary)

**Table:** 

Measurement(s)	Genotype
Technology Type(s)	Report from Literature

## Background & Summary

Chagas disease (CD) is a neglected tropical disease considered a public health concern in Latin America^1^. World Health Organization reports that between 15 and 17 million people get infected, and around 50.000 die out of 100 million people at risk of infection^1–3^. CD is caused by the protozoan parasite *Trypanosoma cruzi*, which is transmitted by kissing bugs, members of the subfamily Triatominae, through their faeces, where the infective forms of the parasite are present^4^. *T. cruzi* is divided into at least seven discrete typing units (DTUs) from TcI to TcVI and Tcbat^5,6^. TcI presents an extensive genetic diversity and is divided according to the transmission cycle in domestic (TcI_Dom_) and sylvatic (TcI_Sylv_) genotypes^7^. The DTUs are commonly associated with epidemiological and ecological scenarios, but no actual associations have been found. Also, some DTUs are related to oral outbreaks in Brazil, Colombia, Venezuela, Bolivia, and French Guiana (TcI, TcV, TcIII, TcIV)^8^. This transmission type makes CD one of the most important foodborne diseases, but the genotypes, epidemiology, and clinical traits remain poorly understood because each geographical zone presents its epidemiological characteristics^8^.

Through the years, many genetic markers and methods have been used to identify, and genotype *T. cruzi* in the lack of a consensus regarding the two aspects previously mentioned, considering that a single genetic marker is not enough to solve the issues of the parasites classification^6^. Even with all the new technologies recently developed, some researchers still choose old-established but more widely used techniques for their investigations, such as band size PCR or RFLP^9–11^. Moreover, considering the vast diversity of the parasite’s DTUs and hosts^6,12–15^, one could imagine the amount of different genetic markers used through time for identification: Spliced-leader intergenic region (SL-IR), microsatellites, kinetoplast DNA (kDNA), heat shock proteins (HSP), 18 S ribosomal RNA subunit (18 S rRNA), cytochrome c oxidase subunit 2 (COII), cytochrome b (Cytb), Glycosylphosphatidylinositol (GPI) and 24Sα rDNA/rDNA subunits (24Sα), to name a few^16–27^. This is a problem for the nomenclature used to classify the DTUs, especially for TcI genotypes, leading to a discussion due to biases of some markers that can be more accurate than others for *T. cruzi* classification^7^. Nevertheless, this debate continues after 12 years that this nomenclature was established. Even with the plethora of studies unveiling the genomic architecture and plasticity of *T. cruzi*^26^.

Some studies describe the geographical distribution of *T. cruzi*’s DTUs to identify epidemiological associations among the genotypes^28,29^. Others focus on the parasite dynamics by performing phylogeographic studies to understand their evolution and the risk of infection to humans^30^, but the last update of DTUs distribution and epidemiology at a continental level was published in 2016 by Izeta-Alberdi and colleagues^30^. Since then, many researchers have studied the parasite and its vectors and hosts using new methodologies. Hence, here we present an update of DTU’s distribution in the Americas, its ecoepidemiological information such as the transmission cycle, hosts, vectors, and the methods and genetic markers used for their identification and genotyping. To accomplish this, we made a systematic review of the literature available on those above using the PubMed database, hoping this can provide insights that lead to the standardization for DTU’s identification to improve future research regarding molecular epidemiology of CD. We published a similar review in 2020, where a database and an interactive map were built and used as a reference for the surveillance of *Leishmania* in the Americas^31^. Therefore, we encourage the scientific community to keep studying the molecular epidemiology of *T. cruzi* for accurate management and surveillance of CD in endemic regions.

## Methods

### Systematic review

For the construction of this metadata, two researchers independently selected the articles following the same instructions as described in the **Information about the databases used as sources** section below; then, a third investigator made another revision to avoid any discrepancy between the results, followed by a three-step debugging process. We extracted the following information from each article: Original code, Sample type, DTU, TcI DTU/genotype, Coordinates (sexagesimal degrees system), Latitude and longitude (decimal degrees system), Country, Continental division, Upper-division (state/province/department/region), Belong to Amazon basin (yes/no), Lower division (department/municipality/community), Local division (municipality/community/village), Date of isolation, Year of isolation/detection, Species of the host, Common name, Source sample, Order of the host, Tribe (only Triatominae), Genus of the host, Cycle (transmission cycle), Genetic marker (for genotyping), Method of identification (of the parasite) and Genes examined. The articles with no complete/clear information regarding sample collection, hosts/vector species, and methods were excluded from the database. Some coordinates were obtained manually using the web page https://www.gps-coordinates.net if the article specified the place-name where the samples were collected. The coordinate system used was WGS84. For the DTUs distribution, we used the software QGIS 3.16 Hannover (https://www.qgis.org/es/site/) to create and edit the maps, and we used the figures from the software R version 3.6.3 with the library “ggplot2”.

### Inclusion and exclusion criteria

Herein, we considered those articles with clinical (Identification method, sample type, and species identified) and complete geographical information. Three languages were considered (Spanish, English, and Portuguese). Information was searched for in the abstract and full article. We excluded articles without the full (.pdf) version or with incomplete information, such as coordinates, source of the sample, vector/hosts from where the parasite was recovered, or reported techniques that did not fulfil the correct identification of the parasite.

### Information about the databases used as sources

For the database construction, we did a PubMed Advanced Search and employed an algorithm using the words “DTU” and “Trypanosoma cruzi” with the Boolean “AND”. The search was done without establishing a time frame. We downloaded the result file and performed a manual depuration to discard articles unrelated to our interests (*i.e*., pharmacological studies, including another trypanosomatids such as *Leishmania spp*, studies related to another hemipteran species). After reading and refining the articles implementing the previously mentioned criteria, we constructed a database by country to debug. Then, those articles were collected in a metadata database. Furthermore, three more independent debugging processes were carried out to check if the articles comply with the required parameters. Finally, a standardization process of the database fields was performed to verify that their content was all in the same format.

### Database fields information

#### Original code

Refers to the code of the samples assigned by the authors of each article.

#### Sample type

This refers to the type of sample from where the parasite was isolated. We considered the following categories: a) Blood, b) Complete Insect, c) Faeces, d) Food, e) Gut, f) Heart, g) Rectal Ampoule, h) Serum, i) Strain, j) Tissues and k) Xenodiagnoses.

#### DTU

This refers to *Trypanosoma cruzi*’s DTU per sample. The categories used were: a) TcI, b) TcII, c) TcIII, d) TcIV, e) TcV, f) TcVI, g) Tcbat, h) TcII or TcV, i)TcII or TcVI, j) TcII to TcVI, k) TcIII or TcIV, l) TcIII to TcVI, m) TcIV or TcVI, n) TcIV to TcVI, o) Unknown).

#### TcI Genotype

Refers to TcI genotyping. They were categorized as follows: a) Sylv (sylvatic), b) Dom (domestic), c) TcIDom/TcISylv and d) Unknown.

#### Source sample

Refers to the organism from where the sample was isolated. We considered the following categories: a) Food, b) Humans, c) Reservoir (non-human animals), and d) Vector.

#### Species

Regarding the species of the host, we divided the database into a) species of the host (complete scientific name), b) common name, c) order of the host, d) tribe (only for Triatominae), e) genus of the host (only Genus) and f) cycle (refers to the transmission cycle of the host: Domestic/Sylvatic/Peridomestic/NA (No data)).

#### Genetic marker

Refers to the nature of the marker: Nuclear, Mitochondrial, Antigen or NA (no data).

#### Method of identification

For optimization, we categorized the tests/methods/techniques as follows: a) Blotting, b) Electrophoretic, c) PCR-based, d) Real-time PCR, e) Sequencing and f) Serologic. Each category includes subcategories described in Table 2.

#### Genes examined

Refers to the genes used in each study for the parasite identification and genotyping (Supplementary Figure 2).

#### Geographical location

We have nine categories in the database: a) Coordinates (in the sexagesimal degree system of coordinates), b) Latitude, c) Longitude, d) Country (where the samples were collected), e) Continental division (South or North America), f) Upper-division (state/province/department/region), g) Belong to the Amazon basin (if the division is in the Amazon basin), h) Lower division (department/province/municipality/community) and i) Local division (municipality/community/village).

#### Dates

Refers to a) Date isolation (Date of the sample collection) and b) Year isolation/detection (Year in which the parasite was detected).

## Data Records

The metadata files are available as a tab file on Universidad del Rosario repository^32^.

**Fig. 1:**
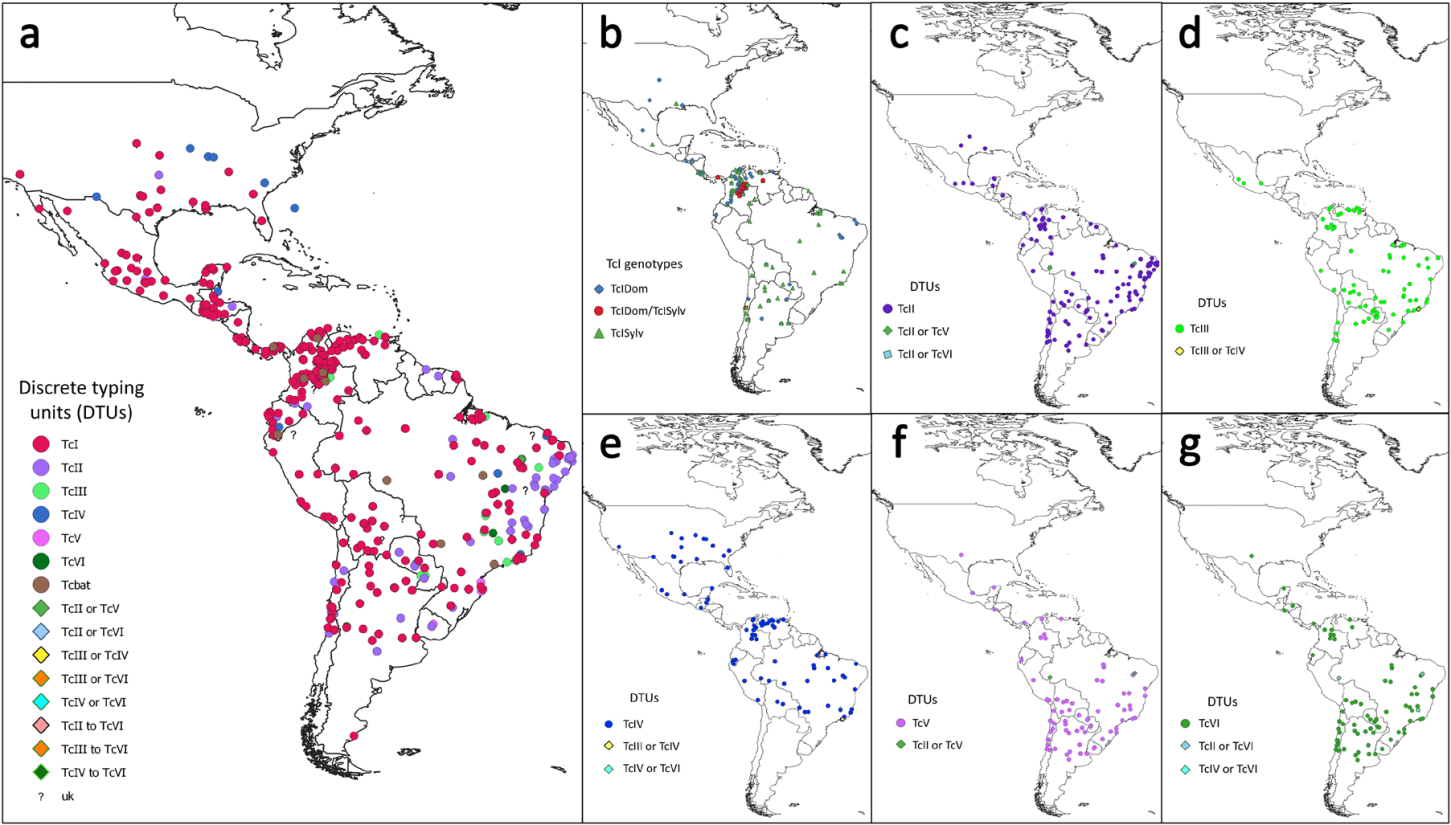
Distribution of *T. cruzi* DTUs in the Americas. (**a**) Consensus map comprising all 15 categories (shown in the legend), (**b**) TcI and its genotypes distribution, (**c**) TcII, (**d**) TcIII, (**e**) TcIV, (**f**) TcV and (**g**) TcVI.

We found a total of 373 articles (data published between 1980 and 2020) from 21 countries in the Americas and two samples from Spain that register the identification of *T. cruzi* DTUs in different hosts and/or vectors (Table 1). Of these, 63.5% of the studies contained Brazil, Colombia, Bolivia, Chile, and Argentina (Table 1). We found a wide distribution of DTUs registered in the continent (Fig. 1a). We also made a distribution map for each DTU where it can be observed that all DTUs are present broadly, especially in South America (Fig. 1). In some studies, DTUs could not be differentiated. Therefore, we opted to put them in a separate category (Fig. 1c,d,e,f,g). Also, it can be noticed that mixed infections between TcI_Dom_ and TcI_Sylv_ were only reported in some countries in the north of South America (Fig. 1b, red points). Moreover, we made an additional map for those categories that comprise a range of DTUs and those that cannot be determined in the studies (Supplementary Figure 4). Finally, in Supplementary Figure 3, there is a distribution map for Tcbat, registered predominantly in Colombia and Brazil (In light of lack of consensus for defining it as a new DTU).

**Table 1:** Summary of the number of studies per sample origin.

Sample origin	Number of studies
Argentina	31
Belize	1
Bolivia	42
Brazil	81
Chile	31
Colombia	52
Costa Rica	3
Ecuador	11
El Salvador	4
French Guiana	5
Guatemala	6
Honduras	5
Mexico	21
Nicaragua	1
Panama	6
Paraguay	16
Peru	14
Surinam	1
USA	20
Uruguay	2
Venezuela	19
Spain*	1
	373

*Samples from patients born in Spain, children of Bolivian immigrants.

**Fig. 2:**
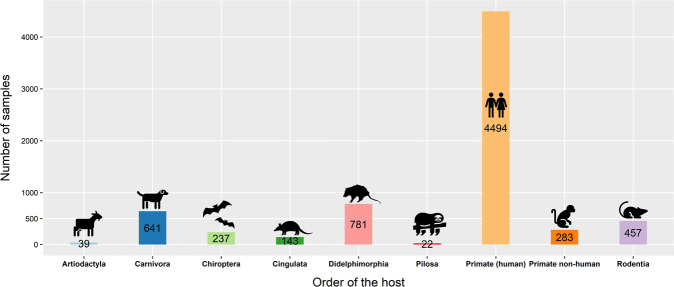
Number of samples obtained from a wide range of hosts (by Order).

**Fig. 3:**
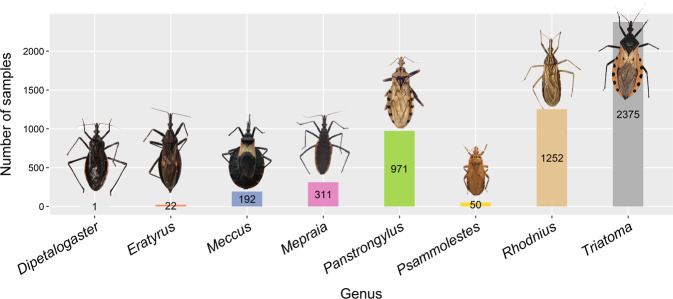
Frequency of samples obtained from different genera of kissing bugs (Hemiptera: Triatominae).

Most of the samples were obtained from Primates (humans), followed by Didelphimorphia, Carnivora, and Rodentia (Fig. 2). Surprisingly, we found two studies where *T. cruzi* was found in food, Açai palm (Arecales) and sugarcane (Poales) (Supplementary Table 1). Moreover, the most common vectors belong to the Genus *Triatoma*, followed by *Rhodnius* and *Panstrongylus* (Fig. 3). Supplementary Figure 1 shows the transmission cycle of the vectors.

**Fig. 4:**
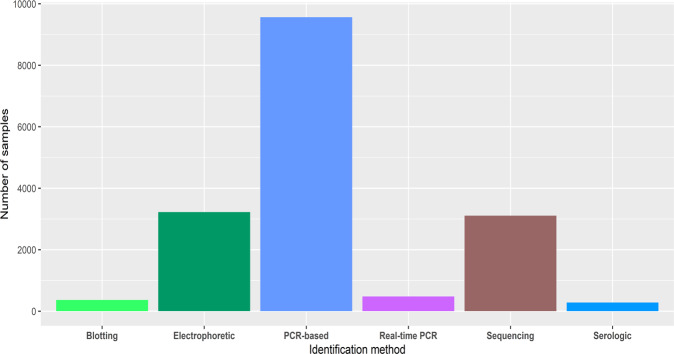
Methods used for the identification and genotyping of *T. cruzi*.

Regarding the methods used for the identification and genotyping of the parasite, we found PCR-based methods as the most widely used, followed by electrophoretic methodologies and sequencing (Fig. 4, Table 2). Furthermore, we counted and manually chose the most common gene algorithms or gene sets used for the identification and genotyping of *Trypanosoma cruzi* (Table 3). Supplementary Figure 2 shows a barplot containing all the different genetic markers used for the above mentioned purpose. In addition, we made a figure that relates the most common genes with the Method of identification/genotyping, where it can be noted that PCR-based methods are the most widely used for most of the genes (Supplementary Figure 5).

**Table 2:** Summary of the different methods used for the identification and genotyping of *T. cruzi*.

Category	Method	Number of samples
Blotting	Western Blot	137
	Southern Blot	226
	TOTAL	363
Electrophoretic	PCR-RFLP	1658
	MLEE	1564
	Size polymorphism of cruzipain CHEF	1
	TOTAL	3223
PCR-based	LSSP-PCR	160
	PCR band size	6181
	RAPD	1395
	Multilocus conventional PCR	154
	Nested PCR	113
	Multiplex PCR	58
	Heminested PCR	76
	PCR-DNA hybridization	1423
	RT-PCR	5
	TOTAL	9565
Real-time PCR	qPCR	375
	Multiplex real-time PCR	79
	Duplex TaqMan qPCR	22
	TOTAL	476
Sequencing	FFLB	47
	MLMT	922
	MLST	657
	Sequencing	1480
	TOTAL	3106
Serologic	ELISA	187
	IFAT	29
	Chagas Sero K-SeT RDT + TSSApep-II/V/VI	65
	TOTAL	281

**Table 3:** Most common gene sets or algorithms used for the identification and genotyping of *T. cruzi*.

Most common gene algorithms	Number of studies
Gene loci/microsatellites/mitochondrial loci/primers/probes	58
24Sα + COII/SL-IR/cytb/GPI/18 S rRNA/A10/HSP60/microsatellites	82
SL-IR	31
SL-IR + GPI/cytb/COII/ND1/18 S/24Sα/kDNA/1f8	27
18 S + SL-IR/COII/A10/cytb/gGAPDH/	46
kDNA maxi/minicircle	32
kDNA maxi/minicircle + SL-IR/cytb/COII/GPI/1f8	8
Total	284

## Technical Validation

Once we obtained the final version of the database, we made the debugging process to assure the correct selection of the data included and their reliability. The first debug was to verify the presence of the parasite (*Trypanosoma cruzi*) in the title or summary of each article. Then, it was made a second debug of the articles but this time considering all the fields information previously described in the methods to define the inclusion or exclusion of the article. Finally, a third debug where a review of the geographical coordinates in detail was conducted. This process allowed us to find any typographical or coordinates errors.

Besides, we decided to treat each sample individually to analyse the DTUs distribution because there were too many categories for this field (DTU). This means that for samples with more than two DTUs (expressed in the database field as *p.e*. TcI/TcII-TcVI), we had to duplicate that sample row or a TcI/TcII/TcV in a single sample, we had to triplicate the row, and so on. To clarify the nomenclature used in the original database, the forward-slash (/) means “and,” and the hyphen (-) means a range (we change it for the expression “to” for the maps). Also, in some studies, the authors report uncertainty between two DTUs (reported as TcIII “or” TcIV). Therefore, we went from 45 DTU categories to 15 (including the unknown) and then put the modified database in the software QGIS to elaborate the map. This new database was used only for this step while keeping the original database for the figures and tables. Finally, due to the high volume of data retrieved, we should divide the original database into four individual archives: hosts, vectors, genes, and methods, each one filtered by the database fields required for the respective analysis. All the plots were created using the packages ggplot2 v3.3.5, circlize v0.4.14 and Biocircos v0.3.4 in RStudio.

## Usage Notes

Due to the high volume of data, we grouped some fields into more general categories. Also, because some geographical coordinates were assigned by searching the place’s name, their precise coordinates may vary.

As explained before, because, in the database, we put many variables in one cell for some fields, we should divide them into individual archives to analyze and make each figure. For the genes examined, used the function filter in Excel to count each gene by selecting the boxes that contained them and put the information in a table along with the marker type (nuclear, mitochondrial, or antigen). The exact process was made for the methods, but in this case, we additionally grouped each Method in a general category to optimize the graphic representation of the results. To make the gene algorithms table, we first looked for the most common genes that we defined as the “principal” ones. Then, we wrote down those other genes that are generally used together with the principal one in the studies and using the filter function, and we checked the boxes containing the principal and complementary genes. Finally, we count the corresponding number of articles (Reference field in the database).

Such as our previous data descriptor of *Leishmania* in the Americas^32^, we now provide an updated *Trypanosoma cruzi* database with ecoepidemiological information to provide a new powerful tool to improve molecular epidemiology research and surveillance in this case for Chagas disease. Contrary to our *Leishmania* data descriptor, here we did not consider a time period for data collection, and also included new categories for hosts like the common name, order and the source (vector, reservoir or human).

We hope this database will be helpful in future research in the field, focusing on achieving a consensus in which are the most reliable genetic markers and methods to identify/genotype *T. cruzi* and keep on trying to understand the transmission dynamics of the parasite.

## Supplementary information

Supplementary table

Supplementary Figures

## Data Availability

We did not use any custom code to process the data described in the manuscript.
