# Supplementary material for: Discrete typing units of Trypanosoma cruzi: Geographical and biological distribution in the Americas
Source: Sci Data. 2022 Jun 24;9:360. doi: 10.1038/s41597-022-01452-w (PMC9232490; doi:10.1038/s41597-022-01452-w)
Supplement: Supplementary file 2 — Supplementary Figures [file 41597_2022_1452_MOESM2_ESM.pdf]

Table of contents

1. Supplementary Figure 1. Number of samples and transmission cycle of the insect vectors and their frequency by genus. **Page 2**

2. Supplementary Figure 2. Barplot comprising the whole array of genetic markers and their type, used for identification and genotyping of *T. cruzi*. **Page 3**

3. Supplementary Figure 3. Tcbat distribution in America. **Page 4**

4. Supplementary Figure 4. Distribution map of the categories “unknown” and DTUs ranges (TcII to TcVI, TcIII to TcVI and TcIV to TcVI). **Page 5**

5. Supplementary Figure 5. Chord diagram that relates the genes with the methods the authors used for the identification/genotyping of the parasite. **Page 6**

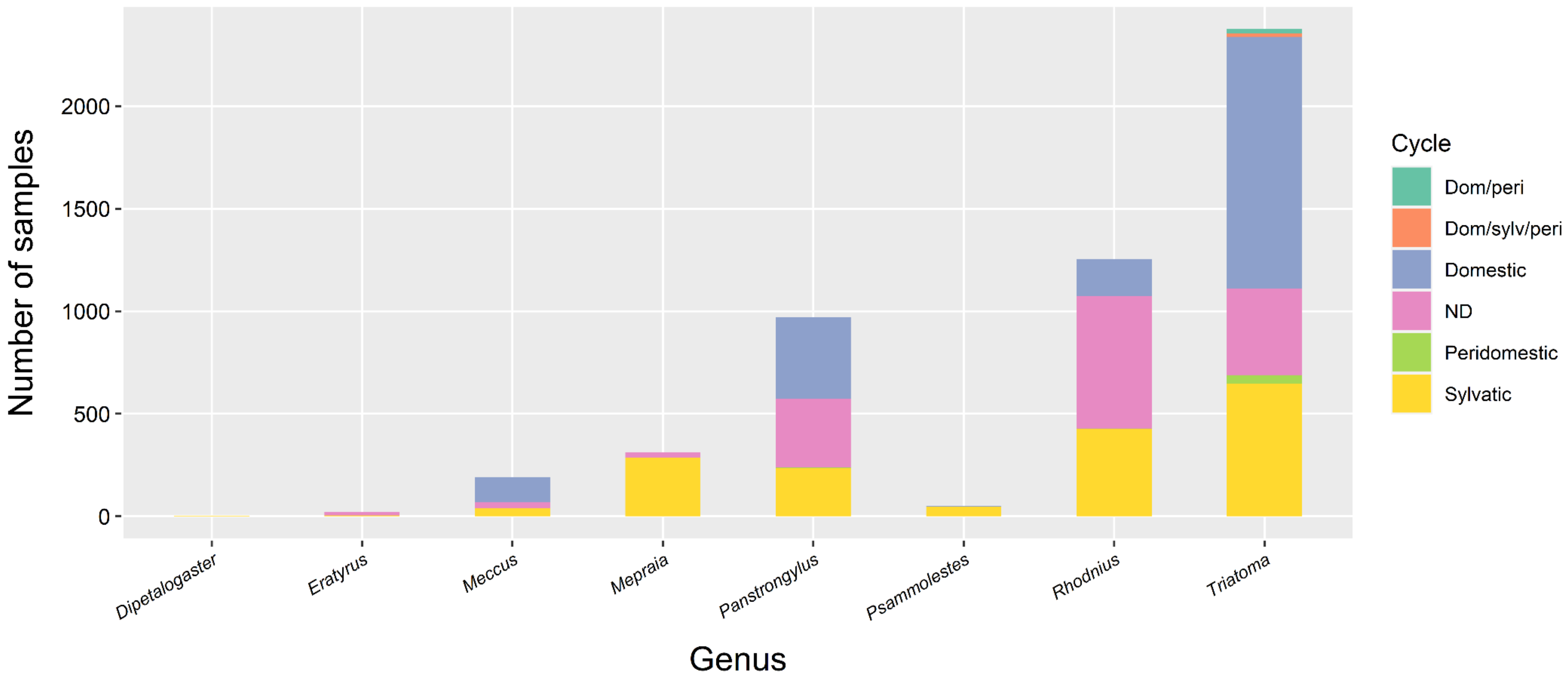

Supplementary Figure 1. Number of samples and transmission cycle of the insect vectors and their frequency by genus.

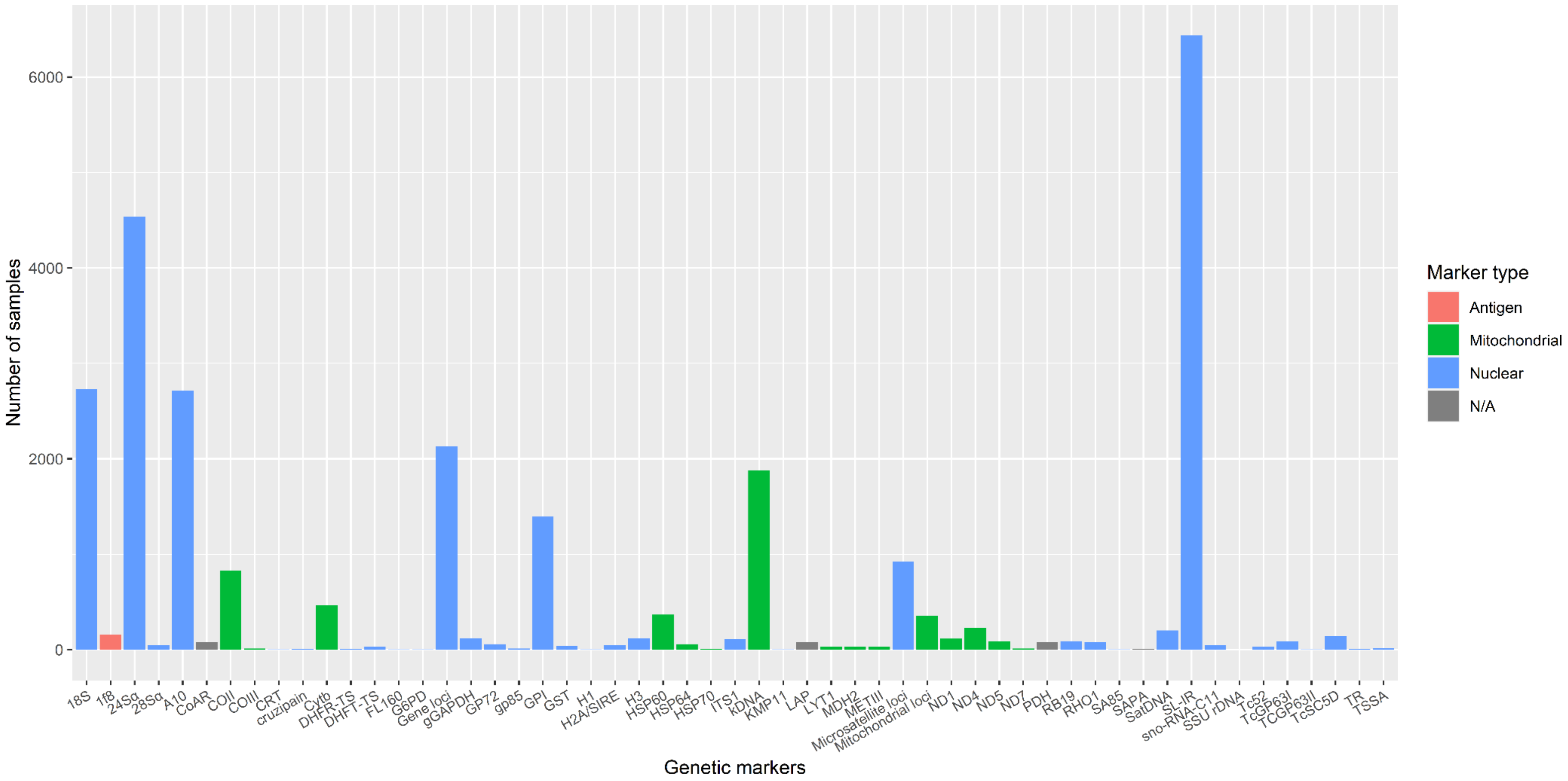

Supplementary Figure 2. Barplot comprising the whole array of genetic markers and their type, used for identification and genotyping of *T. cruzi*.

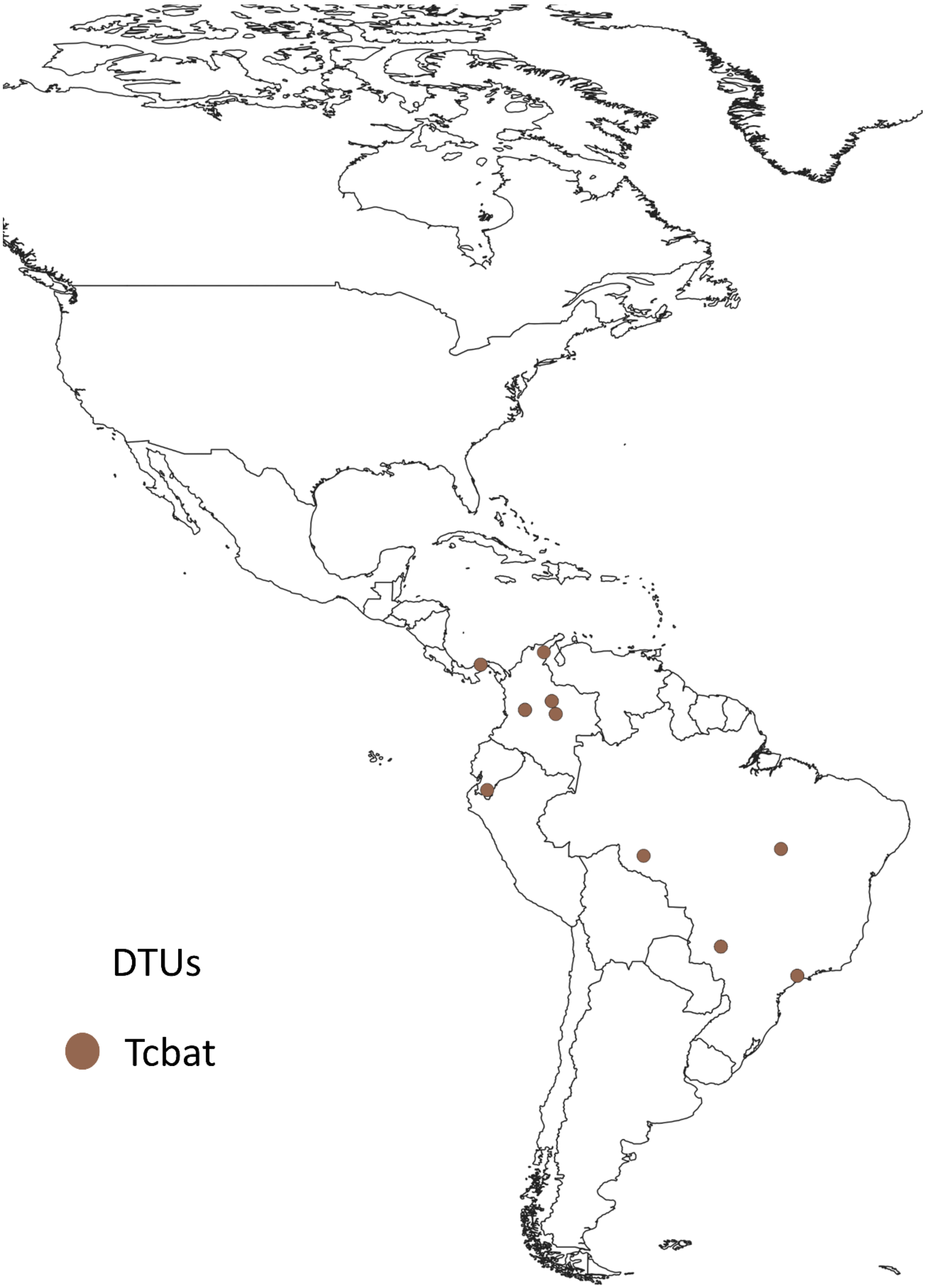

Supplementary Figure 3. Tcbat distribution in America.

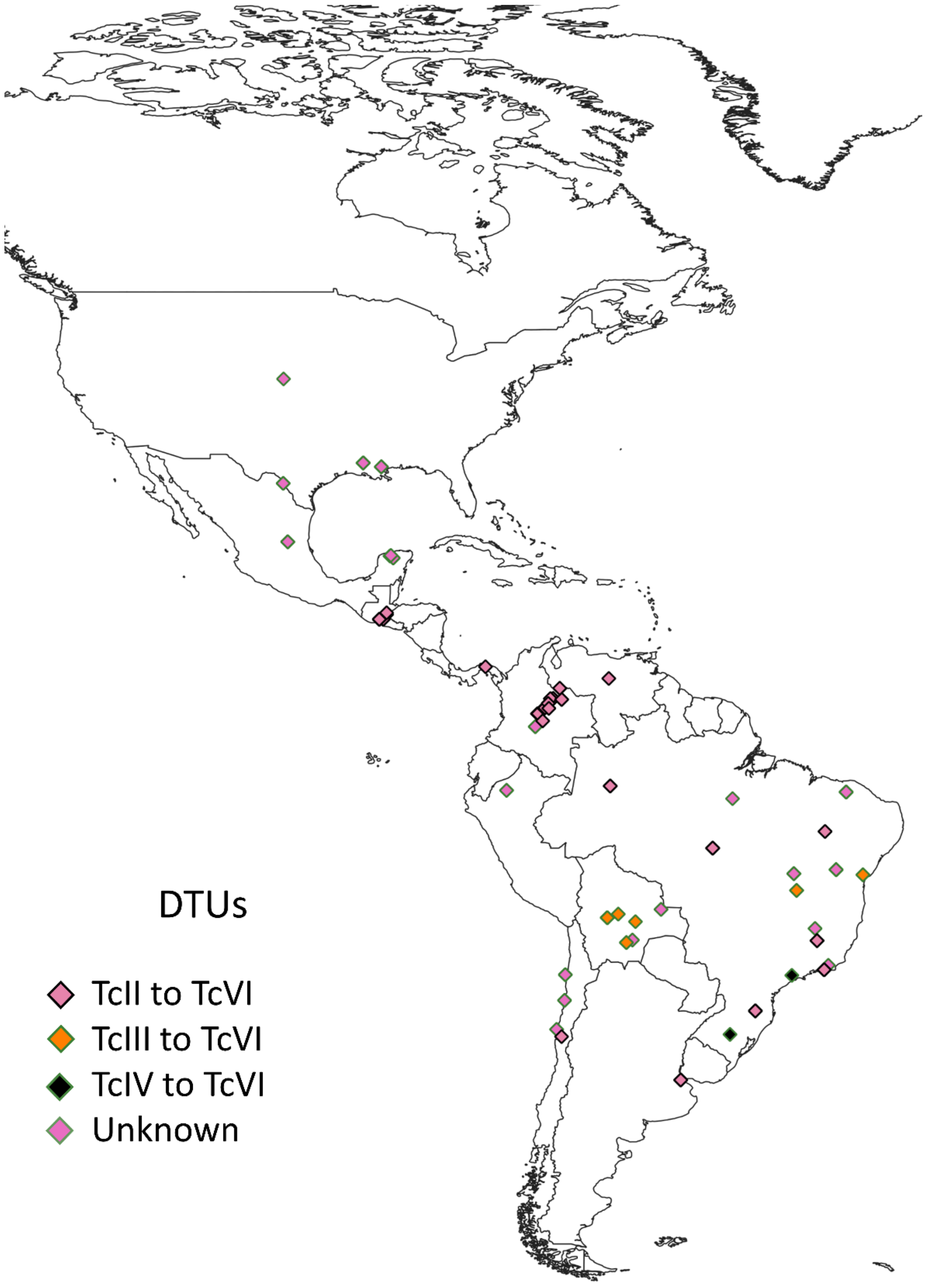

Supplementary Figure 4. Distribution map of the categories "unknown" and DTUs ranges (TcII to TcVI, TcIII to TcVI and TcIV to TcVI).

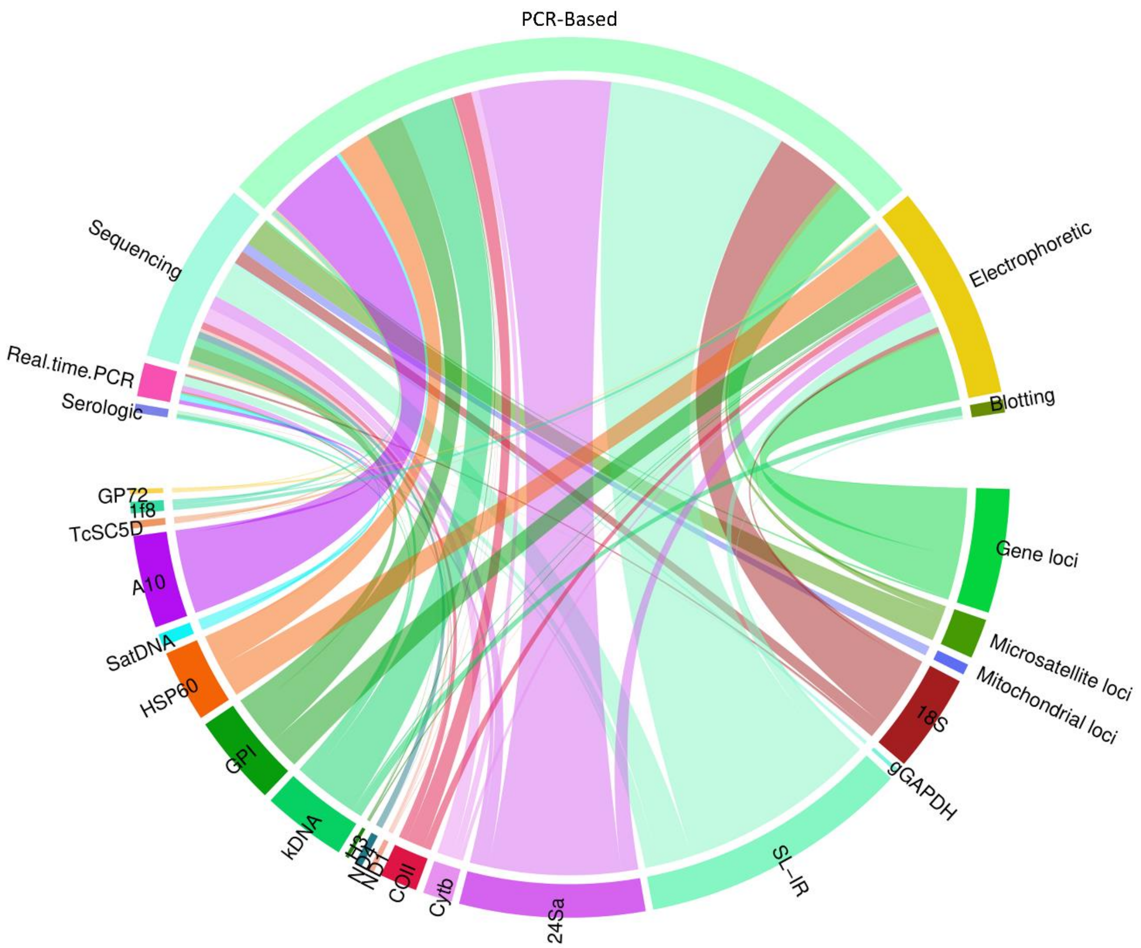

Supplementary Figure 5. Chord diagram that relates the genes with the methods the authors used for the identification/genotyping of the parasite.
